# Supplementary material for: Genetic–Geographic–Chemical Framework of Polyporus umbellatus Reveals Lineage-Specific Chemotypes for Elite Medicinal Line Breeding
Source: J Fungi (Basel). 2026 Jan 3;12(1):39. doi: 10.3390/jof12010039 (PMC12842702; doi:10.3390/jof12010039)
Supplement: Supplementary file 1 [file jof-12-00039-s001.zip › Supplementary Materials/Supplementary material2.pdf]

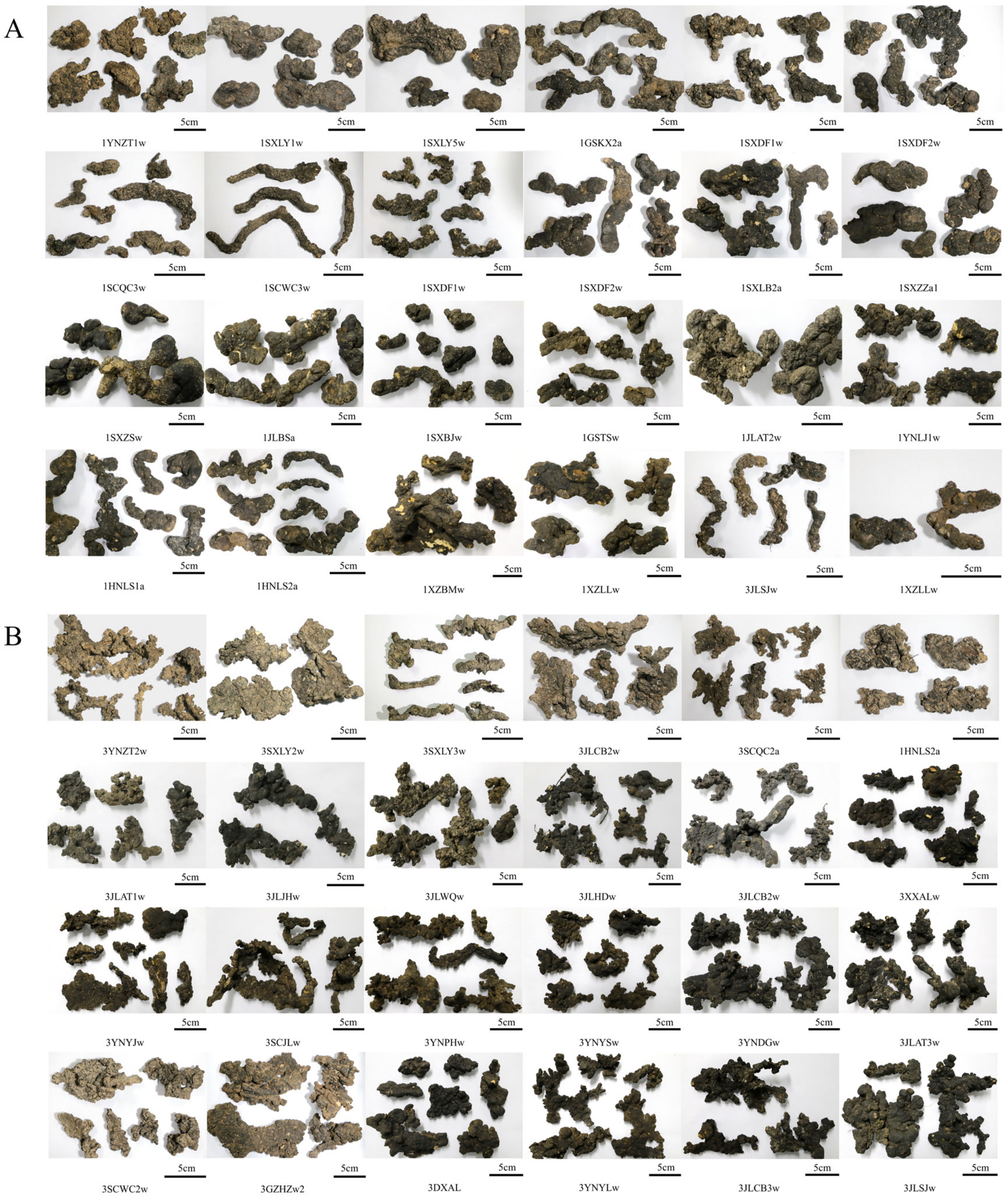

Supplementary Figure S1. Different sclerotium types of *P. umbellatus*: (A)Zhushiling; (B)Jishiling.

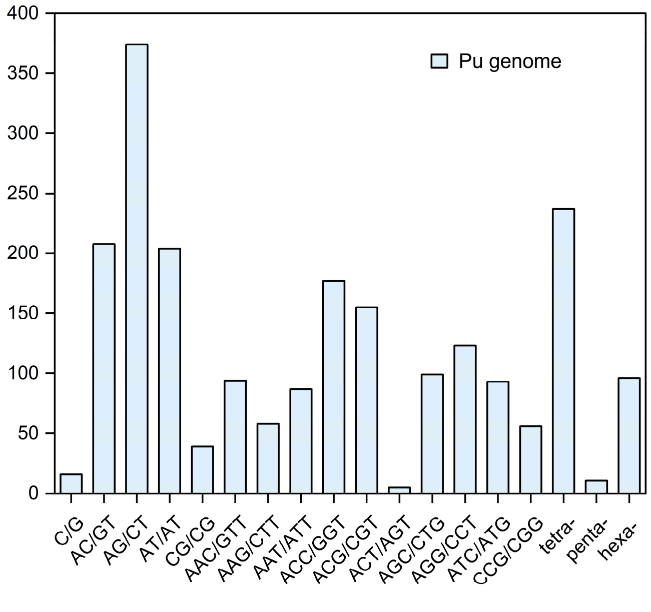

Supplementary Figure S2. Frequency of different types of SSRs in the genome of *P. umbellatus* (including complementary sequences)

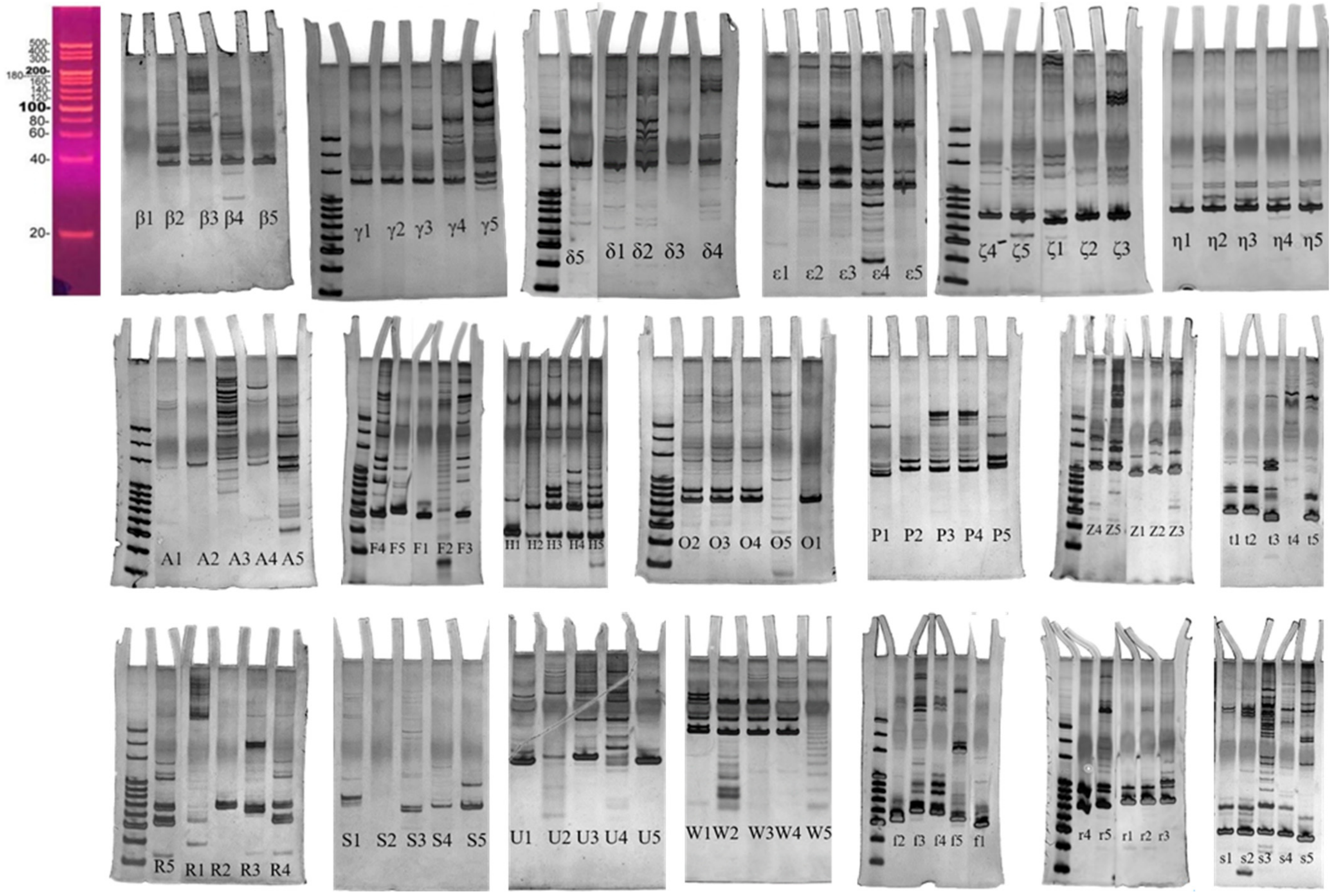

Supplementary Figure S3. Allelic variation in five different *P. umbellatus* germplasms analyzed using 20 SSR primers

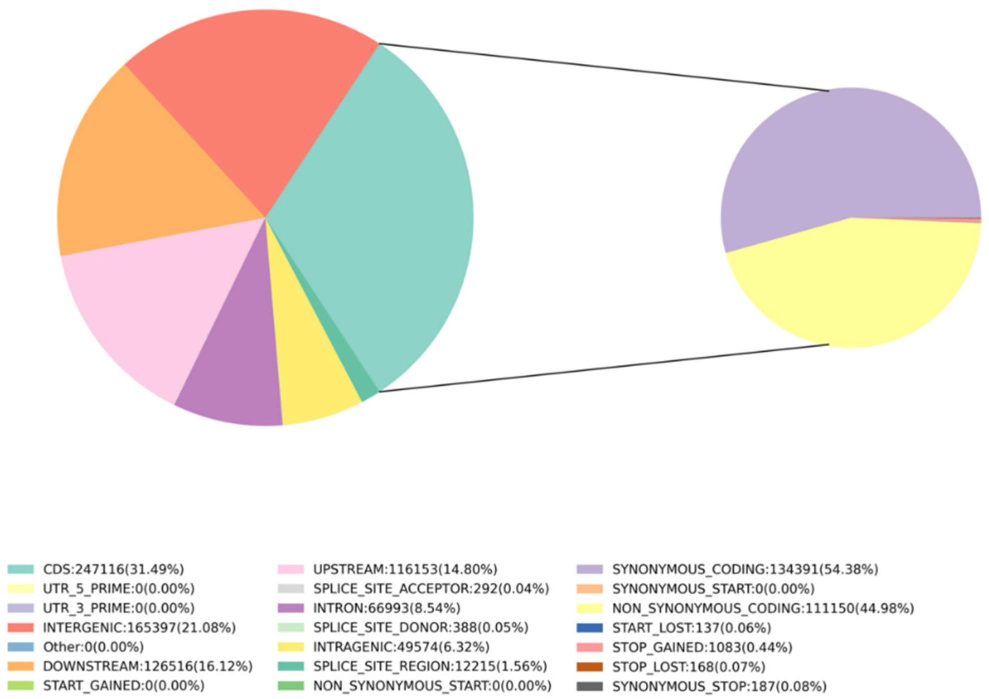

Supplementary Figure S4. Annotation results of SNPs in the *Polyporus umbellatus* genome

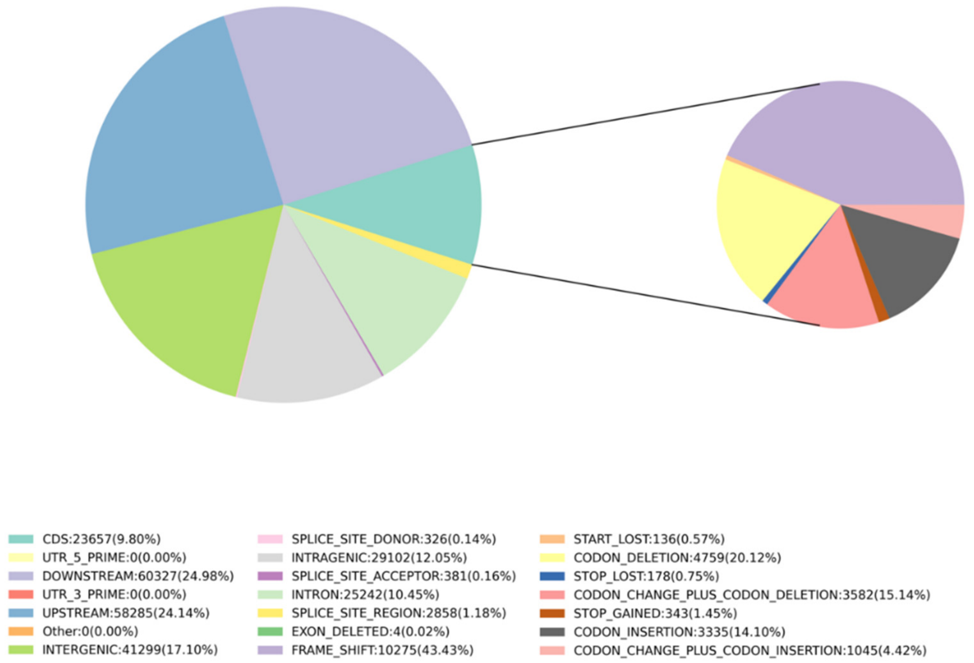

Supplementary Figure S5. Annotation results of Indels in the *Polyporus umbellatus* genome

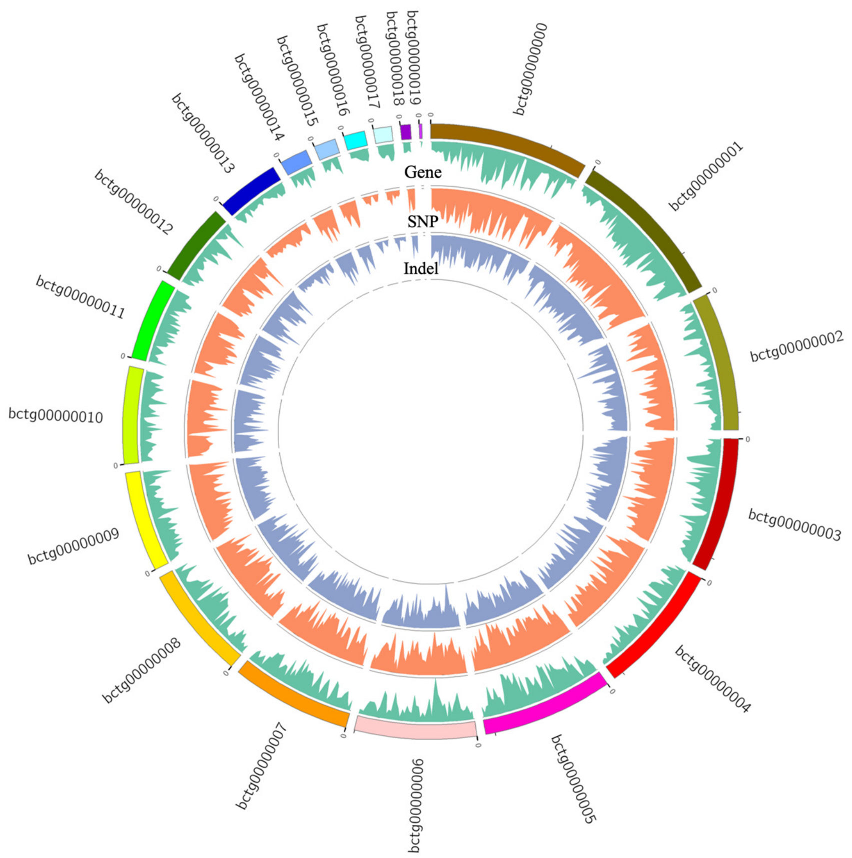

Supplementary Figure S6. Circos plot of SNP and Indel distribution across *Polyporus umbellatus* contigs

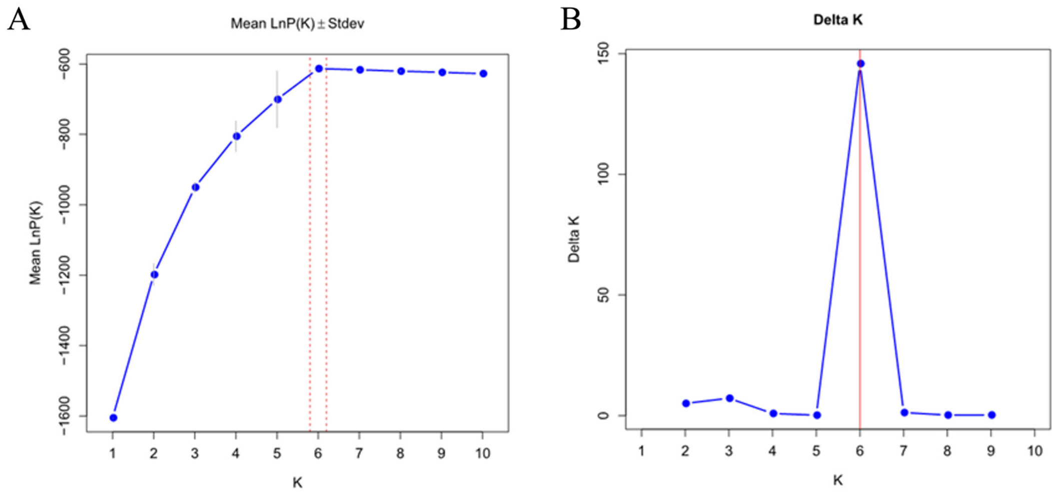

Supplementary Figure S7. Determination of the optimal number of clusters (K) for 53 *P. umbellatus* germplasms based on 19 core SSR markers. (A) Mean log-likelihood probability LnP(K). (B) ΔK statistics identifying K=6 as the optimal cluster number.

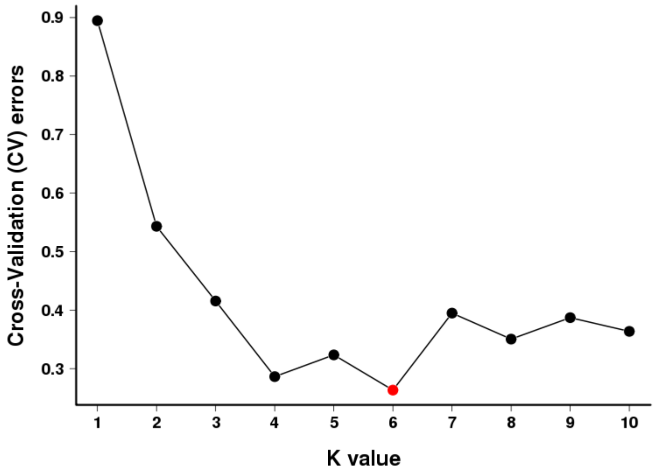

Supplementary Figure S8. Determination of the optimal number of clusters (K) for 47 *P. umbellatus* germplasms based on genomic SNPs. Cross-Validation (CV) error plot identifying K=6 as the optimal cluster number (lowest error).

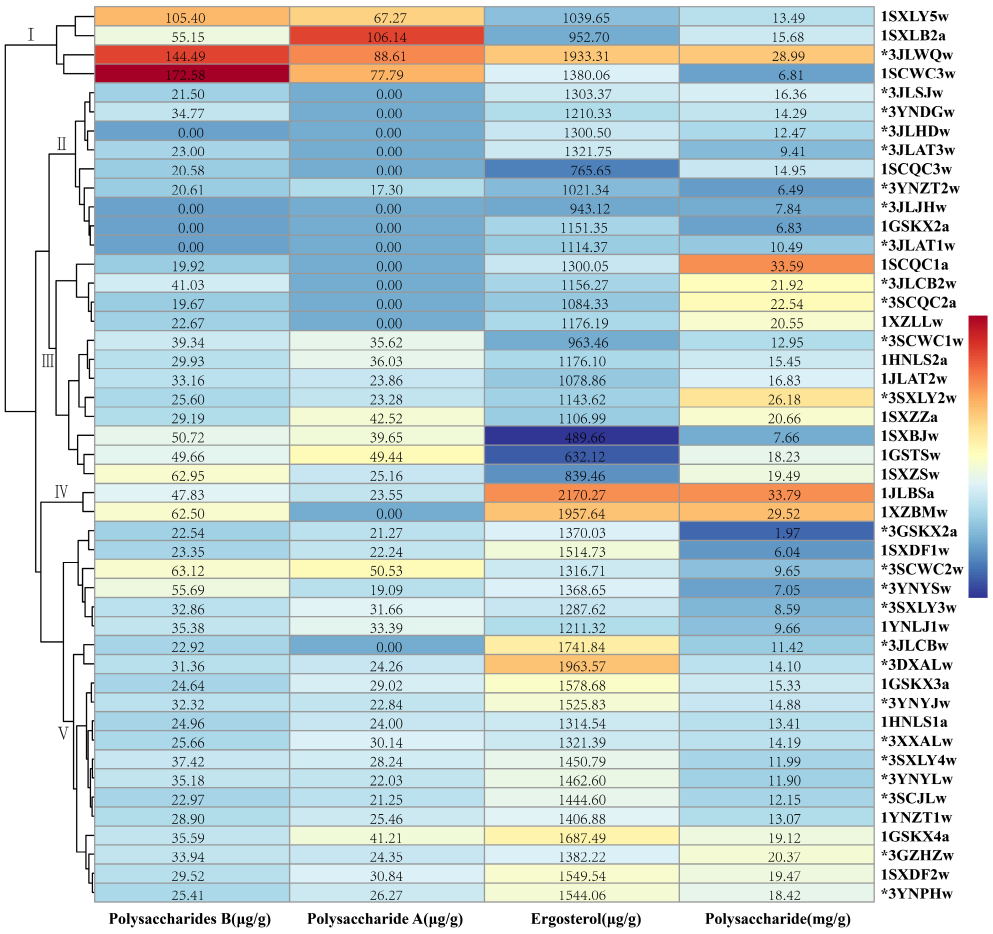

Supplementary Figure S9. Hierarchical clustering heatmap analysis of the four pharmacological components in 47 samples of *P. umbellatus*. The labeled data represent the original measured values prior to Z-score standardization. Asterisks (*) denote Jishiling.
